# Thermomechanical Properties of Polymers and Their Composites with Other Materials: Advances in Thermal and Mechanical Properties of Polymeric Materials (2nd Edition)

**DOI:** 10.3390/ma17020494

**Published:** 2024-01-20

**Authors:** Adam Gnatowski, Agnieszka Kijo-Kleczkowska

**Affiliations:** Faculty of Mechanical Engineering and Computer Science, Czestochowa University of Technology, Dabrowskiego 69, 42-201 Czestochowa, Poland

Progress in the engineering of polymeric materials, including the search for innovative polymer composites with specific properties, has resulted in an expansion of their application areas, especially in the automotive, construction, energy, packaging, and medical industries. The practical use of new polymeric materials requires knowledge of their mechanical, electrical, and thermal properties, as well as recognition of changes in these properties during the operation and destruction of polymers. Due to a growing number of issues related to the processing and recycling of polymeric materials, including waste from energy processes, and concerns about fossil fuel depletion, the use of modern materials in the manufacturing of products is becoming crucial in addressing global environmental pollution.

Observed changes in production technologies, growing demand for manufactured products, the search for modern materials, the pursuit of zero-emission technologies, and the increased energy efficiency of devices, alongside the simultaneous depletion of natural resources, are key elements of sustainable development. In this context, research and testing of new materials are important.

An important aspect of research is the environmental dimension, which includes the combustion/co-combustion of polymers, thermal utilization of polymer waste with energy recovery, and other applications of polymeric materials derived from recycling. 

The Special Issue “Advances in Thermal and Mechanical Properties of Polymeric Materials (2nd Edition)” aims to bring together research on material advances, with a focus on the recycling process and technological progress, including modeling and computer simulation of changes in the materials’ properties.

This work aims to encourage authors to publish papers that deal with the thermomechanical properties of polymers and their composites with other materials, taking into consideration both modeling issues and the practical technological aspects. Examples of relevant research areas and topics are presented below.

Currently, technologies play a crucial role in the manufacturing of composites and polymer blends, the recycling processes, methods of utilizing waste heat in the recycling process, and efficient energy recovery systems. Composite materials are replacing metal materials, as they overcome issues such as corrosion, weight, flexibility, and other challenges. In this context, eco-innovative materials are being developed, and research into hybrid composites reinforced with different fibers is ongoing. Hybrid polymers improve the mechanical properties of their original materials such as stretching, impact resistance, and bending [1,2,3,4]. 

Composites are reinforced with fiber strikethroughs to enhance their thermal properties. Research on the thermal properties of polymer composites is conducted using different methods, including thermogravimetric analysis, differential scanning calorimetry, thermomechanical analysis [5,6,7,8], gas chromatography/mass spectrometry [9], and stereoscopic microscope imaging [10].

Understanding the variability of properties of plastics and composite materials originating from recycling is crucial to facilitating the reintroduction of these materials into handling processes, thereby contributing to the development of a closed-loop economy [11,12,13]. 

Among all recycling techniques for polymer composites, thermal recycling is best suited for recycling carbon fibers and glass fibers. Through thermal recycling, the properties of materials from recycling can match those of the original materials, and the energy used is significantly lower compared to chemical recycling. Another method of plastic recycling involves the direct recovery of energy through incineration or fuel production [14,15,16,17].

The analysis of experimental data and numerical simulations of combustion/co-combustion processes for plastics and fuels indicates possibilities to optimize and conduct these processes, as well as offering further research directions [18,19].

Technological progress in the field of materials, especially in the processing and recycling of composites, poses significant challenges. Through the study of the properties of modern eco-innovative materials, substantial advancements can be achieved, contributing to the development of solutions that enable the attainment of goals in manufacturing products using material recycling and energy recovery. These tasks can be accomplished through collaboration and the exchange of experiences and knowledge.

## References

[1] Kumar G.N., Rajesh K., Rao M.R.D., Bharath K.P.S., Manikanta J.E. (2023). A review on mechanical properties of hybrid polymer composites. Mater. Today Proc..

[2] Arunachalam S.J., Saravanan R. (2023). Study on filler reinforcement in polymer matrix composites—A review. Mater. Today Proc..

[3] Pandit P.P., Liu C., Iacono S., Corti G., Hu Y. (2023). Microstructural Characterization and Property of Carbon Fiber Reinforced High-Density Polyethylene Composites Fabricated by Fused Deposition Modeling. Materials.

[4] Huseynov O., Hasanov S., Fidan I. (2023). Influence of the matrix material on the thermal properties of the short carbon fiber reinforced polymer composites manufactured by material extrusion. J. Manuf. Process..

[5] Yap T., Heathman N., Phillips T., Beaman J., MTehrani M. (2023). Additive Manufacturing of Polyaryletherketone (PAEK) polymers and their composites. Compos. Part B Eng..

[6] Mohd Nasir N.H., Usman F., Woen E.L., Ansari M.N.M., Supian A.B.M., Saloma S. (2023). Microstructural and Thermal Behaviour of Composite Material from Recycled Polyethylene Terephthalate and Fly Ash. Recycling.

[7] Andrzejewski J., Danielak A., Piasecki A., Islam A., Szostak M. (2023). Biocarbon-based sustainable reinforcing system for technical polymers. The structure-properties correlation between polycarbonate (PC) and polybutylene terephthalate (PBT)-based blends containing acrylonitrile-butadiene-styrene (ABS). Sustain. Mater. Technol..

[8] Jalaee A., Kamkar M., French V., Rojas O.J., Foster R.J. (2023). Direct milling: Efficient, facile, and green method for processing fibrillated cellulose/polymeric nanocomposites with boosted thermomechanical and rheological performance. Carbohydr. Polym..

[9] Gnoffo C., Frache A. (2024). Identification of Plastics in Mixtures and Blends through Pyrolysis-Gas Chromatography/Mass Spectrometry. Polymers.

[10] Xanthopoulou E., Chrysafi I., Polychronidis P., Zamboulis A., Bikiaris D.N. (2023). Evaluation of Eco-Friendly Hemp-Fiber-Reinforced Recycled HDPE Composites. J. Compos. Sci..

[11] Jones H., McClements J., Ray D., Hindle C.S., Kalloudis M., Koutsos V. (2023). Thermomechanical Properties of Virgin and Recycled Polypropylene—High-Density Polyethylene Blends. Polymers.

[12] Kim K.-W., Kim D.-K., Han W., Kim B.-J. (2022). Comparison of the Characteristics of Recycled Carbon Fibers/Polymer Composites by Different Recycling Techniques. Molecules.

[13] Costa A.A., Martinho P.G., Barreiros F.M. (2023). Comparison between the Mechanical Recycling Behaviour of Amorphous and Semicrystalline Polymers: A Case Study. Recycling.

[14] Khalid M.Y., Arif Z.A., Ahmed W., Arshad H. (2022). Recent trends in recycling and reusing techniques of different plastic polymers and their composite materials. Sustain. Mater. Technol..

[15] Rajan K.P., Mustafa I., Gopanna A., Thomas S.P. (2023). Catalytic Pyrolysis of Waste Low-Density Polyethylene (LDPE) Carry Bags to Fuels: Experimental and Exergy Analyses. Recycling.

[16] Raveh-Amit H., Lemont F., Bar-Nes G., Klein-BenDavid O., Banano N., Gelfer S., Charvin P., Bin Rozaini T., Sedan J., Rousset F. (2022). Catalytic Pyrolysis of High-Density Polyethylene: Decomposition Efficiency and Kinetics. Catalysts.

[17] Albor G., Mirkouei A., McDonald A.G., Struhs E., Sotoudehnia F. (2023). Fixed Bed Batch Slow Pyrolysis Process for Polystyrene Waste Recycling. Processes.

[18] Ongar B., Beloev H., Georgiev A., Iliev I., Kijo-Kleczkowska A. (2023). Optimization of the Design and Operating Characteristics of a Boiler Based on Three-Dimensional Mathematical Modeling. Bulg. Chem. Commun..

[19] Korobeinichev O., Shmakov A., Paletsky A., Trubachev S., Shaklein A., Karpov A., Sosnin E., Kostritsa S., Kumar A., Shvartsberg V. (2022). Mechanisms of the Action of Fire-Retardants on Reducing the Flammability of Certain Classes of Polymers and Glass-Reinforced Plastics Based on the Study of Their Combustion. Polymers.

